# A phase Ib/IIa trial of 9 repurposed drugs combined with temozolomide for the treatment of recurrent glioblastoma: CUSP9v3

**DOI:** 10.1093/noajnl/vdab075

**Published:** 2021-06-24

**Authors:** Marc-Eric Halatsch, Richard E Kast, Georg Karpel-Massler, Benjamin Mayer, Oliver Zolk, Bernd Schmitz, Angelika Scheuerle, Ludwig Maier, Lars Bullinger, Regine Mayer-Steinacker, Carl Schmidt, Katharina Zeiler, Ziad Elshaer, Patricia Panther, Birgit Schmelzle, Anke Hallmen, Annika Dwucet, Markus D Siegelin, Mike-Andrew Westhoff, Kristine Beckers, Gauthier Bouche, Tim Heiland

**Affiliations:** 1 Department of Neurosurgery, Ulm University Hospital, Ulm, Germany; 2 IIAIGC Study Center, Burlington, Vermont, USA; 3 Institute for Epidemiology and Medical Biometry, Ulm University, Ulm, Germany; 4 Department of Clinical Pharmacology, Ulm University Hospital, Ulm, Germany; 5 Division of Neuroradiology, Department of Diagnostic and Interventional Radiology, Ulm University Hospital, Ulm, Germany; 6 Division of Neuropathology, Department of Pathology, Ulm University Hospital, Ulm, Germany; 7 Central Pharmacy, Ulm University Hospital, Ulm, Germany; 8 Division of Hematology and Oncology, Department of Internal Medicine, Ulm University Hospital, Ulm, Germany; 9 Institute of Experimental Cancer Research, Ulm University Hospital, Ulm, Germany; 10 Department of Pathology and Cell Biology, Columbia University Irving Medical Center, New York, New York, USA; 11 Department of Pediatric and Adolescent Medicine, Basic Research Division, Ulm University Hospital, Ulm, Germany; 12 Anticancer Fund, Brussels, Belgium

**Keywords:** chemotherapy, clinical trial, drug repurposing, glioblastoma, multi-drug combination

## Abstract

**Background:**

The dismal prognosis of glioblastoma (GBM) may be related to the ability of GBM cells to develop mechanisms of treatment resistance. We designed a protocol called Coordinated Undermining of Survival Paths combining 9 repurposed non-oncological drugs with metronomic temozolomide—version 3—(CUSP9v3) to address this issue. The aim of this phase Ib/IIa trial was to assess the safety of CUSP9v3.

**Methods:**

Ten adults with histologically confirmed GBM and recurrent or progressive disease were included. Treatment consisted of aprepitant, auranofin, celecoxib, captopril, disulfiram, itraconazole, minocycline, ritonavir, and sertraline added to metronomic low-dose temozolomide. Treatment was continued until toxicity or progression. Primary endpoint was dose-limiting toxicity defined as either any unmanageable grade 3–4 toxicity or inability to receive at least 7 of the 10 drugs at ≥ 50% of the per-protocol doses at the end of the second treatment cycle.

**Results:**

One patient was not evaluable for the primary endpoint (safety). All 9 evaluable patients met the primary endpoint. Ritonavir, temozolomide, captopril, and itraconazole were the drugs most frequently requiring dose modification or pausing. The most common adverse events were nausea, headache, fatigue, diarrhea, and ataxia. Progression-free survival at 12 months was 50%.

**Conclusions:**

CUSP9v3 can be safely administered in patients with recurrent GBM under careful monitoring. A randomized phase II trial is in preparation to assess the efficacy of the CUSP9v3 regimen in GBM.

**Key Points**

- Glioblastoma escapes pharmacological treatment as a result of cellular heterogeneity and resistance mechanisms.
- A treatment regimen with 9 different drugs (CUSP9v3) in addition to low-dose metronomic temozolomide was devised to tackle this issue.
- CUSP9v3 is safe in patients with recurrent GBM.

**Importance of the Study**

In 2013, we proposed a new concept to treat patients with recurrent GBM called Coordinated Undermining of Survival Paths (CUSP). The CUSP concept attempts to block growth-driving signaling pathways active in GBM. We took advantage of repurposing already-marketed non-oncological drugs and looked at the evidence for their ability to inhibit one or more of the identified GBM growth and cell survival pathways. Including pharmacology, drug interaction, and safety considerations, a list of 9 drugs was proposed to be used with low-dose, continuous temozolomide (CUSP9v3). Here, we report the results from the first clinical trial of CUSP9v3. In 10 patients with recurrent glioblastoma, the regimen was well-tolerated. This work is the first step in establishing that an extensive multi-drug regimen is tolerable and should now be tested for its potential efficacy against GBM.

As of fall 2020, current standard treatment of glioblastoma (GBM) with neurologically safe maximal resection, irradiation and temozolomide leads to progression-free survival (PFS) of 6.7 months, overall survival (OS) of 16.0 months, and 2-year OS of 30.7%.^1^

Recurrence usually takes place within a year after initial treatment. There is no commonly accepted standard of care for recurrent GBM. No regimen has proven to be safe and markedly effective for this condition.^2^

In an attempt to address this unmet need, our group, together with many others, embarked on an exhaustive systematic search for already-marketed non-oncological drugs that might be able to set the stage for temozolomide to be more effective.^3–9^

A complex winnowing process led to the final selection of the 9 drugs of Coordinated Undermining of Survival Paths combining 9 repurposed non-oncological drugs with metronomic temozolomide—version 3 (CUSP9v3) on which we report here the first clinical experiences. Details of that selection process can be found in the background papers.^4,7^ Important criteria for drug selection were 1) robustness of preclinical data on GBM growth inhibition, 2) low side effect burden, 3) clinical familiarity with the drug in its general medicine (non-oncology) role, 4) availability as a generic, non-proprietary drug, and finally 5) lack of predictable serious pharmacological interactions.^4,7^

The drugs of CUSP9v3 with their basic pharmacological attributes are listed in Table 1. Briefly, aprepitant inhibits NK-1 which is a growth-stimulating element in GBM.^10^ The anti-rheumatoid arthritis drug auranofin inhibits thioredoxin reductase, resulting in increased intracellular reactive oxygen species.^11^ The anti-hypertensive captopril reduces invasion, migration and adhesion of GBM cell activity through soluble matrix metalloproteinase (MMP)-2 and MMP-9 inhibition.^12^ The analgesic celecoxib has long been shown to have anticancer properties related to cyclooxygenase-2 inhibition and has demonstrated encouraging results in combination with low-dose temozolomide.^13,14^ The alcohol deterrent disulfiram is consistently cytotoxic to a wide range of cancer cells and is effective against GBM stem cells through aldehyde dehydrogenase inhibition.^15^ The antifungal itraconazole likely exerts its anticancer activity due to its multiple pharmacological effects^16^ with specific data in GBM pointing towards an effect on autophagy.^17^ The antibiotic minocycline has well-characterized neuroprotective effects^18^ and reduces GBM growth and invasion.^19^ The anti-retroviral ritonavir is effective in mouse GBM models with temozolomide by inducing endoplasmic reticulum stress.^20^ Last, the antidepressant sertraline was included for its ability to inhibit P-glycoprotein at the blood-brain barrier^21^ and because of its safe use in GBM patients.^22^ Overall, these drugs were judged to have robust anti-glioma or temozolomide-augmenting effects as well as to meet the criteria 1 to 5 above.

**Table 1.** Drugs Included in CUSP9v3 with Selected Pharmacological and Biological Characteristics

Drug	p450 inhibition	Half-life	Core survival pathway or process targeted	Most frequent side effects according to drug label (in descending order of frequency)
Aprepitant	3A4, 2C9	10 h	NK-1 receptors	Constipation, dyspepsia, fatigue, ALAT increase, decreased appetite, headache, hiccups
Auranofin	None	10 d	Thioredoxin, ROS generation, STAT3	Diarrhea, pruritus, exanthema
Captopril	None	2 h	ACE, AT1 receptors, MMPs	Diarrhea, nausea, dry mouth, constipation, abdominal pain, vomiting, loss of taste, dizziness, dysgeusia, sleep disorders, dyspnea, cough, rash, alopecia, pruritus
Celecoxib	2C9, 3A4	12 h	COX-1 and -2, carbonic anhydrase -2 and -9	Arterial hypertension
Disulfiram	2E1	< 2 h	ALDH, ROS generation	Nausea, vomiting, drowsiness/somnolence
Itraconazole	3A4	19 h	P-gp efflux transporters, BCRP, hedgehog, 5-lipoxygenase	Nausea, abdominal pain, headache
Minocycline	None	10–20 h	Inhibits monocyte, macrophage and microglial contributions to growth	Diarrhea, nausea, vomiting, dyspepsia, flatulence, dizziness, rash, urticaria, pruritus
Ritonavir	3A4	4 h	P-gp efflux transporters (weak), proteasome, Akt, mTOR, cyclin D3	Pancreatitis, diarrhea, nausea, abdominal pain, vomiting, dyspepsia, fatigue, asthenia, flushing, feeling hot, increased amylase, decreased thyroxine, arthralgia, back pain, dizziness, peripheral neuropathy, headache, paresthesia, dysgeusia, oropharyngeal pain, cough, pharyngitis, rash, pruritus
Sertraline	Weak	1 d	Akt, mTOR, TCTP	Diarrhea, nausea, dry mouth, fatigue, dizziness, drowsiness/somnolence, headache, insomnia, ejaculation failure

ACE, angiotensin-converting enzyme; Akt, protein kinase B; ALAT, Alanine aminotransferase; ALDH, aldehyde dehydrogenase; AT1, angiotensin II receptor type 1; BCRP, breast cancer resistance protein; COX, cyclo-oxygenase; MMPs, matrix metalloproteinases; mTOR, mammalian target of rapamycin; NK-1, neurokinin-1; P-gp, P-glycoprotein; ROS, reactive oxygen species; STAT3, signal transducer and activator of transcription 3; TCTP, translationally controlled tumor protein.

CUSP9v3 also comprises low-dose, continuous temozolomide at 20 mg/m^2^ body surface area (BSA) p.o. twice daily, without interruption. This choice was based on past trials of various temozolomide schedules. After evaluating the 15 trials reviewed by Chen et al.^23^ and comparing these to data of Omuro et al., Clarke et al*.,* and Reynés et al.^24–26^ who used temozolomide at 50 mg/m^2^ BSA/day without interruption, that of Stockhammer et al.^14^ who used 20 mg/m^2^ BSA/day and that of Zustovich et al.^27^ who used 40 mg/m^2^ BSA/day, we concluded that any potential advantage of higher dosing was small and offset by a strongly reduced side effect burden associated with a regimen of 50 mg/m^2^ BSA/day or less. Kong et al. reported that temozolomide at the dose of 40 mg/m^2^ BSA/day was well-tolerated even in patients with Karnofsky Performance Status (KPS) < 70%.^28^ We, therefore, chose 20 mg/m^2^ BSA given twice daily, the dose used by Zustovich et al. and Kong et al.^27,28^

Although the safety profile of each drug of the CUSP9v3 protocol is well-known, safety concerns may arise due to the risk of drug-drug interactions at the pharmacodynamic (eg, in form of additive toxicity) or pharmacokinetic level (with effects on metabolism or elimination, requiring dose adjustments or drug pausing). A database search prior to study initiation showed that clinically relevant interactions between CUSP9v3 drugs are expected to occur mainly due to CYP3A inhibition by itraconazole (strong), ritonavir (strong), and aprepitant (moderate). The unusual risks of using 10 daily drugs over a protracted period were partially offset by the good safety profile of each when used as a single drug and the intensity of our monitoring of patients.

We report here the results of the first trial of the CUSP9v3 regimen for recurrent or progressive GBM.

## Patients and Methods

### Study Design

This is a phase Ib/IIa trial examining the safety of the CUSP9v3 regimen combined with temozolomide in patients with recurrent or progressive GBM. The primary endpoint was dose-limiting toxicity (DLT), and secondary endpoints were best tumor response, PFS, and OS. Dose-limiting toxicity was defined as either any unmanageable grade 3–4 toxicity at the end of the second treatment cycle or inability to receive at least 7 of the 10 drugs, all of them being given at ≥ 50% of the target doses, at the end of the second treatment cycle. Best tumor response was defined as the best therapeutic effect recorded from the start of the treatment until the last follow-up according to Response Assessment in Neuro-Oncology (RANO) criteria.^29^

Overall survival was defined as the time in months between the CUSP9v3 induction cycle start date and the date of last follow-up or death of any cause, whichever came first. Patients alive at the time of the last follow-up were censored.

Progression-free survival was defined as the time between the CUSP9v3 induction cycle start date and the date of the last follow-up, progression according to RANO criteria, or death of any cause, whichever came first. Patients with no progression and alive at the time of the last follow-up were censored.

This study was approved by the institutional review board of Ulm University Hospital (approval number 112/16) and the German competent authority Bundesinstitut für Arzneimittel und Medizinprodukte (BfArM; reference number 4041326) and registered at clinicaltrials.gov (NCT02770378).

### Sample Size

A sample size of 10 patients was selected to assess the primary endpoint. In this population, we expected a true rate of DLT of 40%. Sequential boundaries were used to monitor the DLT rate with accrual to be halted if excessive numbers of DLTs were seen. A Pocock-type stopping boundary yielded a probability of crossing the boundaries of maximally 10% when the actual rate of DLT was equal to the expected rate of 40%.^30^ The boundaries are described in Supplementary Material 1.

### Patients

Eligible patients were adults with histologically confirmed GBM and recurrent or progressive disease according to RANO criteria. In 3 cases, study inclusion was allowed based on early recurrence that had not yet met minimal RANO requirements (ie, 10 mm × 10 mm diameters) but was judged as recurrence by external radiologists and confirmed by the trial’s neuroradiologist (Be.S.). Patients with prior low-grade glioma were eligible if the malignant transformation to GBM was histologically confirmed. Additional key eligibility criteria were: no more than 3 prior episodes of tumor progression, KPS of at least 70%, stable steroid dose for at least 1 week prior to the start of study treatment, sufficient interval since last treatment (at least 4 weeks for systemic treatment or surgery, at least 12 weeks for radiotherapy) and no known contraindication to any of the CUSP9v3 drugs.

### Treatment Regimen

Treatment initiation encompassed the addition of the 9 drugs to uninterrupted temozolomide as depicted in Supplementary Material 2 and comprised an induction cycle with 2 phases: a low-dose drug-by-drug addition phase followed by an up-dosing phase. Patients were hospitalized during the drug-by-drug addition phase, which lasted 18 days, to monitor tolerability and drug-drug interactions.

In summary, as schematically depicted in Supplementary Material 2, the treatment started with temozolomide (20 mg/m^2^ BSA b.i.d.) and aprepitant (80 mg q.d.) on day 1, followed by the addition of 1 drug every 2 days (day 3, day 5 etc.) at the low-dose level. The last drug (auranofin) was added on day 17. On day 19, the up-dosing phase started with the dose of only one drug being increased every 2 days. The doses of temozolomide and aprepitant remained unchanged, 7 drugs were up-dosed only once and 1 drug (ritonavir) was up-dosed twice.

After reaching target doses of all drugs, the regimen remained unchanged until side effects mandated dose modifications and/or drug pausing or until tumor progression occurred. While the study was locked after the last recruited patient had completed 12 months of treatment, patients without tumor progression continued to receive the CUSP9v3 regimen beyond that point.

### Safety and Dose Modifications

In addition to the potential drug-drug interactions to be monitored, we assessed the cumulative toxicity of the regimen. Employing the summary of product characteristics of each of the 10 drugs, we were able to identify the side effects most likely to occur during treatment. By developing a simple algorithm based on the frequency of each side effect (from very common [occurs in ≥ 1/10 patients] to very rare [occurs in < 1/10 000 patients]), we elaborated a strategy for dose modifications, dose re-escalations, and on-hold rules. For instance, in case of fatigue, no action would be taken for grade 1 or grade 2 fatigue. If grade 3 fatigue occurred, the first drug on a hierarchical list specific for fatigue was to be held until grade 2 or lower was reached and resumed at the same level. Additional drugs were to be held in the absence of resolution of symptoms.

During the induction cycle and the first 2 treatment cycles, adjustments (dose reductions and drug pausing) were allowed to accommodate the patients’ individual tolerability of the regimen. These modifications were discussed by a team comprising a neurosurgeon (M.-E.H.), an oncologist (R.M.-S.), a pharmacologist (O.Z.), and a psychiatrist (R.E.K.). For each patient, the regimen tolerated at the time of completion of the second treatment cycle (around day 90) was used to assess the primary endpoint.

### Response Assessment

Response to study treatment was determined by neurological examination and contrast-enhanced magnetic resonance imaging (MRI) using the RANO criteria. Assessment was done at week 6, week 10 and then every 8 weeks. We used the best overall response, *i.e.*, the best response recorded from the start of the treatment, as a secondary endpoint.

### Statistical Analysis

Study data were analyzed by means of descriptive methods using frequencies (absolute and relative values) for categorical data as well as median and range for metric data. The Kaplan-Meier method was used to calculate PFS and OS. The median PFS and OS, respectively, are presented along with their corresponding 95% confidence intervals (CI). All analyses were performed using SAS (version 9.4, www.sas.com) and R (version 3.5.2, www.r-project.org).

## Results

### Patients Characteristics

Ten patients were included between August 2016 and April 2018. A total of 12 patients were screened. One patient could not be included because of high serum transaminases and one because of acute deep vein thrombosis. Demographic characteristics of the 10 included patients are presented in Table 2.

**Table 2.** Demographic Characteristics of the 10 Patients Included in the CUSP9v3 Trial

Characteristics	*N* (%)
Sex	
Male	6 (60)
Female	4 (40)
Median age at diagnosis in years (range)	41 (25–60)
Type of GBM	
Primary	8 (80)
Secondary	2 (20)
KPS at baseline	
100	4 (40)
90	2 (20)
80	1 (10)
70	3 (30)
Recurrence/progression at inclusion	
First	6 (60)
Second	4 (40)
Median time between first diagnosis and start of CUSP9v3 in months	16
Tumor location at time of study entry	
Frontal lobe	2 (20)
Temporal lobe	2 (20)
Parietal lobe	1 (10)
Disseminated—basal ganglia	1 (10)
Disseminated—midbrain and brainstem	2 (20)
Disseminated—callosal	2 (20)
Initial extent of resection	
Gross total	7 (70)
Subtotal	3 (30)
*MGMT* promoter status	
Hypermethylated	6 (60)
Non-hypermethylated	4 (40)
*IDH1/2* status	
Mutated	2 (20)
Wild-type	8 (80)
Prior therapies	
Surgery	10 (100)
Radiotherapy	10 (100)
Temozolomide	10 (100)
Bevacizumab	1 (10)
Tetrahydrocannabinol	1 (10)
TTFields^TM^	1 (10)

### Safety

The defined Pocock-type safety boundaries for stopping the trial were not crossed at any time. Nine patients completed at least 2 treatment cycles. At the end of the second treatment cycle, no patient had experienced any unmanageable grade 3–4 toxicity, and all patients had received at least 7 of the 10 drugs, given at ≥ 50% of the target doses. The primary endpoint was therefore met. Most frequently paused were ritonavir (for ataxia and fatigue), temozolomide (for diarrhea, nausea and laboratory abnormalities), captopril (for diarrhea and nausea), and itraconazole (for diarrhea and laboratory abnormalities) while ritonavir (for gait disturbance) and captopril (for fatigue) were most frequently dose-reduced.

All patients experienced at least one adverse event (AE) of any grade (Table 3) and 7 patients (70%) experienced at least one grade 3–4 AE, including 2 with at least one grade 4 AE. Ten AEs occurred in 5 or more patients. These AEs are presented in Table 3 with their grades and the drug(s) to which the respective AE was most likely related. All grade 3–4 AEs are presented in Table 4, grouped by classes, and listed with the drug(s) to which the AE was attributed or deemed possibly related. For all central nervous system AEs, no direct relationship could be established between the suspected drug and the respective AE because of the underlying disease.

**Table 3.** Number of Patients Experiencing at Least One Adverse Event (AE) by Grade and Related Drugs, for the Most Frequent AEs

	All grades	Grade 3–4	Grade 1	Grade 2	Grade 3	Grade 4	Drug to which AE was most frequently attributed (or possibly related)
All AEs	10	7	10	9	7	2	
Most frequent AEs							
Fatigue	9	1	2	6	1	-	Temozolomide
Nausea	9	1	4	4	1	-	Temozolomide (all 10 drugs deemed possibly related for at least 1 patient)
Headache	8	1	5	2	1	-	Sertraline
Seizure	6	2	2	2	2	-	Sertraline
Lymphocyte count decrease	6	6	-	-	5	1	Temozolomide
Diarrhea	5	-	4	1	-	-	Temozolomide (followed by minocycline and sertraline)
Dysgeusia	5	-	5	-	-	-	Sertraline
Gait disturbance	5	3	-	2	3	-	Sertraline
Bradycardia	5	-	5	-	-	-	Captopril
Tremor	5	-	4	1	-	-	Sertraline

**Table 4.** List of all Grade 3–4 Adverse Events (AEs) and Related Drugs

AE	N	Drug(s) to which AE was attributed	Drug(s) to which AE was possibly related
Liver and pancreatic enzymes			
ALAT increased	4	Temozolomide	Minocycline, celecoxib, captopril, itraconazole, ritonavir, auranofin
ASAT increased	1	Temozolomide	Aprepitant, minocycline, celecoxib, captopril, ritonavir
GGT increased	1	Temozolomide	Minocycline, celecoxib, captopril, ritonavir
Lipase increased	1	-	Temozolomide, itraconazole
Hematology			
Lymphocyte count decreased	8	Temozolomide, itraconazole, ritonavir, auranofin	Minocycline, celecoxib, captopril, itraconazole, ritonavir, auranofin
White blood cell decreased	1	-	Temozolomide, minocycline, celecoxib, itraconazole, ritonavir, auranofin
Platelet count decreased	3	Temozolomide	
Central nervous system			
Aphasia	1	-	-
Ataxia	2	-	Sertraline, celecoxib
Confusion	1	-	Sertraline, captopril
Edema, cerebral	2	-	-
Gait disturbance	5	-	Sertraline
Headache	1	-	-
Hypoglossal nerve disorder	1	-	-
Psychosis	1	-	-
Pyramidal tract syndrome	2	-	-
Seizure	2	-	Sertraline
Vagus nerve disorder	1	-	-
Gastro-intestinal			
Dysphagia	1	-	-
Nausea	1	-	All 10 drugs
Other			
Fatigue	1	-	Temozolomide
Hypotension	1	-	Captopril
Lung infection	1	-	Temozolomide
Muscle weakness lower limb	1	-	-
Thromboembolic event	1	-	-

ALAT, Alanine aminotransferase; ASAT, Aspartate aminotransferase: GGT, Gamma-glutamyltransferase.

All but one of the AEs attributed to CUSP9v3 drugs ceased upon pre-specified, targeted dose reduction or drug pausing within a range of 0 to 4 weeks (median 2 weeks; median number of drug modifications necessary to revert an AE: 1.5). None of the AEs that had ceased upon dose reduction or drug pausing recurred after the suspected drugs were reinstated.

### Efficacy

Best overall response was stable disease (SD) in 6 patients and progressive disease (PD) in 4 patients (Table 5). Median duration of response was 8 (range 1–11) months in responders at the time of data lock. For 5 patients, SD was ongoing at the time of reporting. Three patients developed no detectable tumor on MRI during study treatment but would not be assigned “complete response” according to RANO because their tumor was “non-measurable” on MRI (ie, had maximal diameters of < 10 mm × 10 mm) at study entry. However, early recurrence had been initially diagnosed by external radiologists and was confirmed by the trial’s neuroradiologist (Be.S.) on the basis of a > 25% increase of “non-measurable” disease according to RANO.

**Table 5.** Tumor Characteristics, Prior Treatment and Outcomes on CUSP9v3 for Each Patient

Patient ID	Age at inclusion (years)	KPS at inclusion (%)	Prior treatment besides standard of care *	*MGMT* promoter status/*IDH1/2* mutation	Best response	PFS (months)	Vital status at data lock
1	31	100	Re-resection	Methylated/mutated	SD	29	Alive
2	48	80	Re-resection, re-RT	Methylated/wild-type	PD	2	Deceased
3	60	70	Bevacizumab	Non-methylated/wild-type	PD	0	Deceased
4	53	100	-	Methylated/wild-type	SD	21	Alive
5	41	100	Re-resection, tetrahydrocan nabinol	Methylated/wild-type	SD	21	Alive
6	41	70	-	Methylated/wild-type	PD	0	Deceased
7	30	90	-	Non-methylated /wild-type	SD	17	Alive
8	47	70	Re-resection	Methylated/wild-type	PD	2	Deceased
9	25	90	Re-resection	Non-methylated /mutated	SD	3	Deceased
10	27	100	TTFields^TM^	Non-methylated /wild-type	SD	12	Alive

* All 10 patients had been treated with surgery, chemo-radiotherapy and adjuvant temozolomide.

ID, identification; IDH1/2, isocitratdehydrogenase 1 or 2 gene; KPS, Karnofsky Performance Score; MGMT, O6-methylguanine-DNA methyltransferase; PD, progressive disease; PFS, progression-free survival; RT, radiotherapy; SD, stable disease.

**Figure 1.**
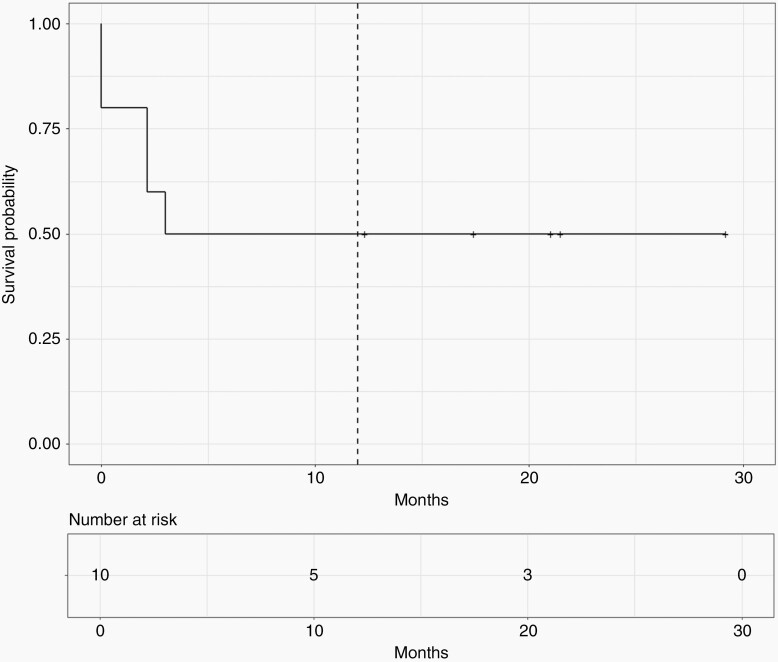
Progression-free survival since CUSP9v3 start.

**Figure 2.**
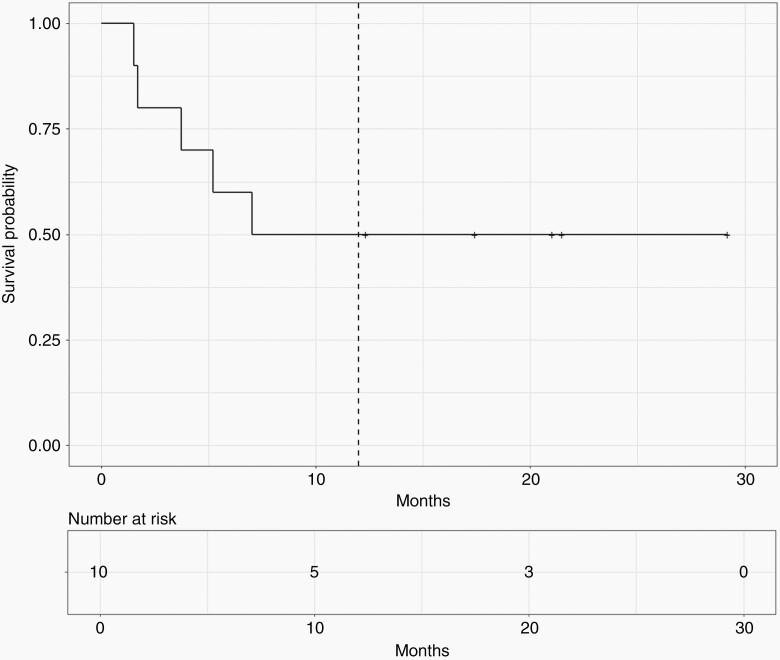
Overall survival since CUSP9v3 start.

Progression-free survival and OS are presented in Figure 1 and Figure 2, respectively. Both PFS and OS at 12 months were 50% with large confidence intervals because of the small sample (95% CI, 27–93%). Table 3 shows each patient’s individual PFS together with their disease characteristics and treatment prior to study entry.

## Discussion

In this phase Ib/IIa trial in recurrent or progressive GBM, we found that 9 carefully selected non-oncological repurposed drugs together with twice daily 20 mg/m^2^ BSA temozolomide was safe and generally well-tolerated if individual dose adjustments were performed. CUSP9v3 is well-enough tolerable to be started in outpatients and to be fully introduced over a shorter time period than the 35 days that we used. The most frequent AEs are not expected to cause management problems as they are well-known to physicians treating patients with recurrent GBM.

Other trials in pediatric and adult high-grade glioma had reported the safety of various multi-agent regimens combining chemotherapy with repurposed drugs, using a range of 4–7 agents.^31–33^ Here we show that it is possible to combine 9 repurposed drugs given a careful evaluation of potential drug-drug interactions and cumulative toxicity. Knowing that many non-oncological drugs target pathways relevant to GBM, precision oncology approaches could expand their armamentarium by evaluating non-cancer drugs and combining them with classical cancer drugs.^34,35^ Such was recently reported in a trial in diffuse intrinsic pontine glioma.^36^ As strategies targeting cell membrane marker-defined glioblastoma cells may be limited,^37^ CUSP9v3 is consciously intended as a biomarker-independent approach.

During the protocol development for this trial, a hierarchical drug list had been developed (based on AE information contained in each drug’s summary of product characteristics) that correlated AEs to ranked sequences of drugs to be halved in dose or paused until pre-specified lower Common Toxicity Criteria of Adverse Events grades were reached. If that was not the case within 3 days, the next drug on the hierarchical list was halved in dose or paused etc. This strategy proved successful in managing AEs.

Noteworthy, the 3 AEs most frequently observed in this trial were nausea, headache and fatigue. While a causal relationship between the CUSP9v3 drugs and these cardinal AEs cannot be excluded, these symptoms may also be caused by the underlying disease itself and/or its primary treatment, temozolomide.

While the trial was not designed to assess the efficacy of the CUSP9v3 regimen, we observed that 5 patients progressed quickly, dying within a range of 1.5–7 months. The 5 other patients did well on treatment, all 5 having a PFS of 12 or more months (range 12–29 months at the time of data lock). In recurrent GBM, single-agent trials have reported PFS at 6 months of up to 20–30%.^38,39^ The rate of patients being alive and progression-free at 6 months has been suggested as an appropriate surrogate endpoint for predicting OS.^40^

However, the small number of patients prevents any interpretation about the efficacy of the CUSP9v3 regimen. Another limitation is that the trial did not require histopathological confirmation of recurrence prior to study entry. Therefore, despite radiological judgement and reasonable time periods between the completion of radio- and chemotherapy on the one hand and the beginning of the study on the other hand (12 and 4 weeks, respectively), patients with a favorable course could have had pseudo-progression upon starting CUSP9v3. This issue of pseudo-progression is inherent to non-randomized trials in the recurrent setting.

One hypothesis that is supported by the pronounced dichotomy of response to CUSP9v3 is that CUSP9v3 may be more effective in patients with slower proliferating tumors and/or lower tumor burden, suggesting that this regimen may have a role in a prophylactic maintenance setting after first-line treatment.

Drug repurposing represents a large source of therapeutic options in cancer.^41,42^ In GBM in particular, notably 76 repurposed drugs recently were reported as potentially useful.^34^ The selection of the 9 drugs to be included in CUSP9v3 was a long iterative process within a conceptual framework that considered the specific and relevant preclinical, pharmacological, and empirical features of each drug in addition to the 5 criteria listed in the Introduction. It should not be assumed that combining other repurposed non-cancer drugs will automatically yield similar results; other regimens may prove more or less toxic or more or less effective.

In the common aggressive cancers, and especially in GBM, phenotypic spatial and temporal heterogeneity, in both stem and non-stem cell subsets, is a dynamic process responding to treatment interventions and driven further by hypoxia.^43–45^ In addition, GBM may be considered a collection of mutually interacting, mutually supporting cellular subpopulations^46^ demanding the use of a multi-drug combination to achieve prolonged treatment response.

## Conclusions

We report here the first clinical trial of the CUSP9v3 regimen in recurrent or progressive GBM. The treatment regimen was safe under clinical, laboratory and electrocardiogram monitoring. A multicenter randomized controlled phase II/III trial is in preparation to assess the efficacy of the CUSP9v3 regimen in GBM.

This pilot study met its primary endpoint with no unpredicted side effects resulting from the combination of drugs, and there was a signal of a potential positive effect of CUSP9v3 that should be tested in future trials.

## Supplementary Material

vdab075_suppl_Supplementary_Materials

## Notes

**Authorship Statement**. M.E.H., R.E.K., G.B., and K.B. conceived the study. M.E.H., T.H., G.K.-M., C.S., K.Z., Z.E., P.P., A.D., T.H., O.Z., Be.S., A.S., L.M., L.B., Bi.S., M.D.S., and M.-A.W. were involved in the conduct of the trial. B.M., T.H., M.E.H., and R.E.K. analyzed and interpreted the data. M.E.H., R.E.K., G.B., and T.H. wrote the manuscript. All authors reviewed the manuscript.

## References

[1] Lakomy R , Kazda T, Selingerova I, et al. Real-world evidence in glioblastoma: stupp’s regimen after a decade. Front Oncol. 2020;10:840. 32719739 10.3389/fonc.2020.00840 PMC7348058

[2] Mandel JJ , Yust-Katz S, Patel AJ, et al. Inability of positive phase II clinical trials of investigational treatments to subsequently predict positive phase III clinical trials in glioblastoma. Neuro Oncol. 2018;20(1):113–122. 29016865 10.1093/neuonc/nox144 PMC5761583

[3] Skaga E , Skaga IØ, Grieg Z, Sandberg CJ, Langmoen IA, Vik-Mo EO. The efficacy of a coordinated pharmacological blockade in glioblastoma stem cells with nine repurposed drugs using the CUSP9 strategy. J Cancer Res Clin Oncol. 2019;145(6):1495–1507. 31028540 10.1007/s00432-019-02920-4 PMC6527541

[4] Kast RE , Boockvar JA, Brüning A, et al. A conceptually new treatment approach for relapsed glioblastoma: coordinated undermining of survival paths with nine repurposed drugs (CUSP9) by the International Initiative for Accelerated Improvement of Glioblastoma Care. Oncotarget. 2013;4(4):502–530. 23594434 10.18632/oncotarget.969 PMC3720600

[5] Serafin MB , Bottega A, da Rosa TF, et al Drug repositioning in oncology. Am J Ther. 2021;28(1):e111–e117. 31033488 10.1097/MJT.0000000000000906

[6] Halatsch ME , Kast RE, Dwucet A, et al. Bcl-2/Bcl-xL inhibition predominantly synergistically enhances the anti-neoplastic activity of a low-dose CUSP9 repurposed drug regime against glioblastoma. Br J Pharmacol. 2019;176(18):3681–3694. 31222722 10.1111/bph.14773 PMC6715605

[7] Kast RE , Karpel-Massler G, Halatsch ME. CUSP9* treatment protocol for recurrent glioblastoma: aprepitant, artesunate, auranofin, captopril, celecoxib, disulfiram, itraconazole, ritonavir, sertraline augmenting continuous low dose temozolomide. Oncotarget. 2014;5(18):8052–8082. 25211298 10.18632/oncotarget.2408 PMC4226667

[8] Purow B . Repurposing existing agents as adjunct therapies for glioblastoma. Neurooncol Pract. 2016;3(3):154–163. 31386097 10.1093/nop/npv041 PMC6668279

[9] Patil VM , Bhelekar A, Menon N, et al. Reverse swing-M, phase 1 study of repurposing mebendazole in recurrent high-grade glioma. Cancer Med. 2020;9(13):4676–4685. 32400117 10.1002/cam4.3094 PMC7333848

[10] Akazawa T , Kwatra SG, Goldsmith LE, et al. A constitutively active form of neurokinin 1 receptor and neurokinin 1 receptor-mediated apoptosis in glioblastomas. J Neurochem. 2009;109(4):1079–1086. 19519779 10.1111/j.1471-4159.2009.06032.x PMC2696067

[11] Madeira JM , Gibson DL, Kean WF, Klegeris A. The biological activity of auranofin: implications for novel treatment of diseases. Inflammopharmacology. 2012;20(6):297–306. 22965242 10.1007/s10787-012-0149-1

[12] Rooprai HK , Kandanearatchi A, Maidment SL, et al. Evaluation of the effects of swainsonine, captopril, tangeretin and nobiletin on the biological behaviour of brain tumour cells in vitro. Neuropathol Appl Neurobiol. 2001;27(1):29–39. 11299000 10.1046/j.0305-1846.2000.00298.x

[13] Koki AT , Masferrer JL. Celecoxib: a specific COX-2 inhibitor with anticancer properties. Cancer Control. 2002;9(2 Suppl.):28–35. 11965228 10.1177/107327480200902S04

[14] Stockhammer F , Misch M, Koch A, et al. Continuous low-dose temozolomide and celecoxib in recurrent glioblastoma. J Neurooncol. 2010;100(3):407–415. 20446016 10.1007/s11060-010-0192-y

[15] Wang W , Darling JL. How could a drug used to treat alcoholism also be effective against glioblastoma? Expert Rev Anticancer Ther. 2013;13(3):239–241. 23477507 10.1586/era.12.169

[16] Pantziarka P , Sukhatme V, Bouche G, Meheus L, Sukhatme VP. Repurposing Drugs in Oncology (ReDO) – itraconazole as an anti-cancer agent. Ecancermedicalscience. 2015;9:521. 25932045 10.3332/ecancer.2015.521 PMC4406527

[17] Liu R , Li J, Zhang T, et al. Itraconazole suppresses the growth of glioblastoma through induction of autophagy: involvement of abnormal cholesterol trafficking. Autophagy. 2014;10(7):1241–1255. 24905460 10.4161/auto.28912 PMC4203550

[18] Garrido-Mesa N , Zarzuelo A, Gálvez J. Minocycline: far beyond an antibiotic. Br J Pharmacol. 2013;169(2):337–352. 23441623 10.1111/bph.12139 PMC3651660

[19] Hu F , Ku MC, Markovic D, et al. Glioma-associated microglial MMP9 expression is upregulated by TLR2 signaling and sensitive to minocycline. Int J Cancer. 2014;135(11):2569–2578. 24752463 10.1002/ijc.28908 PMC4519695

[20] Rauschenbach L , Wieland A, Reinartz R, et al. Drug repositioning of antiretroviral ritonavir for combinatorial therapy in glioblastoma. Eur J Cancer. 2020;140:130–139. 33091717 10.1016/j.ejca.2020.09.017

[21] O’Brien FE , Dinan TG, Griffin BT, Cryan JF. Interactions between antidepressants and P-glycoprotein at the blood-brain barrier: clinical significance of in vitro and in vivo findings. Br J Pharmacol. 2012;165(2):289–312. 21718296 10.1111/j.1476-5381.2011.01557.x PMC3268186

[22] Caudill JS , Brown PD, Cerhan JH, Rummans TA. Selective serotonin reuptake inhibitors, glioblastoma multiforme, and impact on toxicities and overall survival: the Mayo Clinic experience. Am J Clin Oncol. 2011;34(4):385–387. 20859197 10.1097/COC.0b013e3181e8461a

[23] Chen C , Xu T, Lu Y, Chen J, Wu S. The efficacy of temozolomide for recurrent glioblastoma multiforme. Eur J Neurol. 2013;20(2):223–230. 22680781 10.1111/j.1468-1331.2012.03778.x

[24] Omuro A , Chan TA, Abrey LE, et al. Phase II trial of continuous low-dose temozolomide for patients with recurrent malignant glioma. Neuro Oncol. 2013;15(2):242–250. 23243055 10.1093/neuonc/nos295 PMC3548585

[25] Clarke JL , Iwamoto FM, Sul J, et al. Randomized phase II trial of chemoradiotherapy followed by either dose-dense or metronomic temozolomide for newly diagnosed glioblastoma. J Clin Oncol. 2009;27(23):3861–3867. 19506159 10.1200/JCO.2008.20.7944 PMC2727290

[26] Reynés G , Balañá C, Gallego O, Iglesias L, Pérez P, García JL. A phase I study of irinotecan in combination with metronomic temozolomide in patients with recurrent glioblastoma. Anticancer Drugs. 2014;25(6):717–722. 24322542 10.1097/CAD.0000000000000059

[27] Zustovich F , Landi L, Lombardi G, et al Sorafenib plus daily low-dose temozolomide for relapsed glioblastoma: a phase II study. Anticancer Res. 2013;33(8):3487–3494. 23898124

[28] Kong DS , Lee JI, Kim WS, et al. A pilot study of metronomic temozolomide treatment in patients with recurrent temozolomide-refractory glioblastoma. Oncol Rep. 2006;16(5):1117–1121. 17016602

[29] Wen PY , Macdonald DR, Reardon DA, et al Updated response assessment criteria for high-grade gliomas: response assessment in neuro-oncology working group. J Clin Oncol. 2010;28(11):1963–1972. 20231676 10.1200/JCO.2009.26.3541

[30] Ivanova A , Qaqish BF, Schell MJ. Continuous toxicity monitoring in phase II trials in oncology. Biometrics. 2005;61(2):540–545. 16011702 10.1111/j.1541-0420.2005.00311.x

[31] Maraka S , Groves MD, Mammoser AG, et al Phase 1 lead-in to a phase 2 factorial study of temozolomide plus memantine, mefloquine, and metformin as postradiation adjuvant therapy for newly diagnosed glioblastoma. Cancer. 2019;125(3):424–433. 30359477 10.1002/cncr.31811 PMC7180384

[32] Robison NJ , Campigotto F, Chi SN, et al. A phase II trial of a multi-agent oral antiangiogenic (metronomic) regimen in children with recurrent or progressive cancer. Pediatr Blood Cancer. 2014;61(4):636–642. 24123865 10.1002/pbc.24794 PMC4285784

[33] Zapletalova D , André N, Deak L, et al. Metronomic chemotherapy with the COMBAT regimen in advanced pediatric malignancies: a multicenter experience. Oncology. 2012;82(5):249–260. 22538363 10.1159/000336483

[34] Basso J , Miranda A, Sousa J, Pais A, Vitorino C. Repurposing drugs for glioblastoma: from bench to bedside. Cancer Lett. 2018;428:173–183. 29729291 10.1016/j.canlet.2018.04.039

[35] Pantziarka P , Bouche G, André N. “Hard” drug repurposing for precision oncology: the missing link? Front Pharmacol. 2018;9:637. 29962954 10.3389/fphar.2018.00637 PMC6010551

[36] Mueller S , Jain P, Liang WS, et al. A pilot precision medicine trial for children with diffuse intrinsic pontine glioma – PNOC003: a report from the Pacific Pediatric Neuro-Oncology Consortium. Int J Cancer. 2019;145(7):1889–1901. 30861105 10.1002/ijc.32258

[37] Kersch CN , Claunch CJ, Ambady P, et al. Transcriptional signatures in histologic structures within glioblastoma tumors may predict personalized drug sensitivity and survival. Neurooncol Adv. 2020;2(1):vdaa093. 32904984 10.1093/noajnl/vdaa093 PMC7462280

[38] Sepúlveda-Sánchez JM , Vaz MÁ, Balañá C, et al. Phase II trial of dacomitinib, a pan-human EGFR tyrosine kinase inhibitor, in recurrent glioblastoma patients with EGFR amplification. Neuro Oncol. 2017;19(11):1522–1531. 28575464 10.1093/neuonc/nox105 PMC5737732

[39] Silvani A , De Simone I, Fregoni V, et al.; Italian Association of Neuro-Oncology. Multicenter, single arm, phase II trial on the efficacy of ortataxel in recurrent glioblastoma. J Neurooncol. 2019;142(3):455–462. 30726533 10.1007/s11060-019-03116-z

[40] Han K , Ren M, Wick W, et al. Progression-free survival as a surrogate endpoint for overall survival in glioblastoma: a literature-based meta-analysis from 91 trials. Neuro Oncol. 2014;16(5):696–706. 24335699 10.1093/neuonc/not236 PMC3984546

[41] Pantziarka P , Verbaanderd C, Sukhatme V, et al. ReDO_DB: the repurposing drugs in oncology database. Ecancermedicalscience. 2018;12:886. 30679953 10.3332/ecancer.2018.886 PMC6345075

[42] Pantziarka P , Verbaanderd C, Huys I, Bouche G, Meheus L. Repurposing drugs in oncology: from candidate selection to clinical adoption. Semin Cancer Biol. 2021;68:186–191. 31982510 10.1016/j.semcancer.2020.01.008

[43] Dirkse A , Golebiewska A, Buder T, et al. Stem cell-associated heterogeneity in glioblastoma results from intrinsic tumor plasticity shaped by the microenvironment. Nat Commun. 2019;10(1):1787. 30992437 10.1038/s41467-019-09853-z PMC6467886

[44] Grimes DR , Jansen M, Macauley RJ, Scott JG, Basanta D. Evidence for hypoxia increasing the tempo of evolution in glioblastoma. Br J Cancer. 2020;123(10):1562–1569. 32848201 10.1038/s41416-020-1021-5 PMC7653934

[45] Lam KHB , Valkanas K, Djuric U, Diamandis P. Unifying models of glioblastoma’s intratumoral heterogeneity. Neurooncol Adv. 2020;2(1):vdaa096. 33005895 10.1093/noajnl/vdaa096 PMC7513885

[46] Guo M , van Vliet M, Zhao J, et al. Identification of functionally distinct and interacting cancer cell subpopulations from glioblastoma with intratumoral genetic heterogeneity. Neurooncol Adv. 2020;2(1):vdaa061. 32642713 10.1093/noajnl/vdaa061 PMC7309246

