# Supplementary material for: A phase Ib/IIa trial of 9 repurposed drugs combined with temozolomide for the treatment of recurrent glioblastoma: CUSP9v3
Source: Neurooncol Adv. 2021 Jun 24;3(1):vdab075. doi: 10.1093/noajnl/vdab075 (PMC8349180; doi:10.1093/noajnl/vdab075)
Supplement: vdab075_suppl_Supplementary_Materials [file vdab075_suppl_supplementary_materials.docx]

Supplementary Table 1

Pocock-type stopping boundaries for a true dose-limiting toxicity rate of 40% and a 10% desired probability of early stopping in 10 patients

| Number of patients, n | 1 | 2 | 3 | 4 | 5 | 6 | 7 | 8 | 9 | 10 |
| --- | --- | --- | --- | --- | --- | --- | --- | --- | --- | --- |
| Boundary, b_n_ | - | - | - | 4 | 5 | 5 | 6 | 6 | 7 | 7 |

Supplementary Figure 1


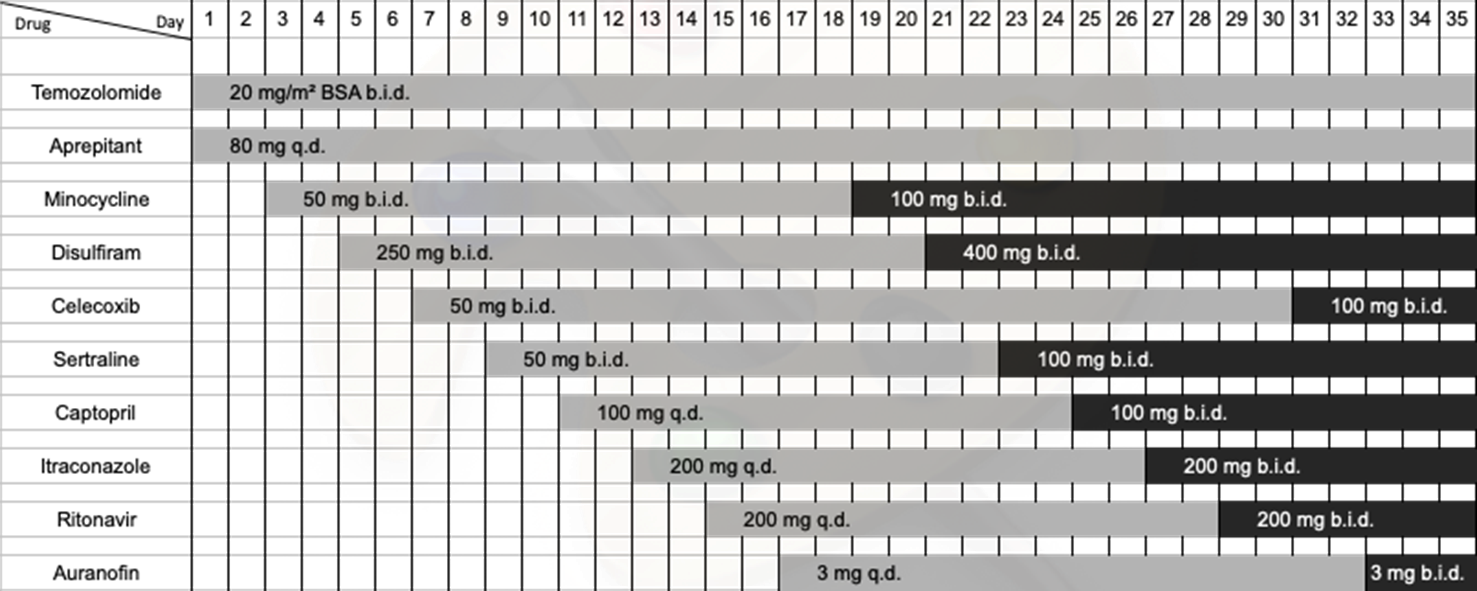


Progressive addition schedule of the CUSP9v3 drugs

BSA: body surface area; b.i.d.: twice a day; q.d.: once a day.
